# Effects of Mesenchymal Stem Cell-Derived Exosomes on Autoimmune Diseases

**DOI:** 10.3389/fimmu.2021.749192

**Published:** 2021-09-27

**Authors:** Ziwei Shen, Wei Huang, Jun Liu, Jie Tian, Shengjun Wang, Ke Rui

**Affiliations:** ^1^ Department of Laboratory Medicine, Affiliated Hospital of Jiangsu University, Zhenjiang, China; ^2^ Department of Immunology, Jiangsu Key Laboratory of Laboratory Medicine, School of Medicine, Jiangsu University, Zhenjiang, China

**Keywords:** mesenchymal stem cells, exosomes, immunoregulation, therapy, autoimmune diseases

## Abstract

Recent years, the immunosuppressive properties of mesenchymal stem cells (MSCs) have been demonstrated in preclinical studies and trials of inflammatory and autoimmune diseases. Emerging evidence indicates that the immunomodulatory effect of MSCs is primarily attributed to the paracrine pathway. As one of the key paracrine effectors, mesenchymal stem cell-derived exosomes (MSC-EXOs) are small vesicles 30-200 nm in diameter that play an important role in cell-to-cell communication by carrying bioactive substances from parental cells. Recent studies support the finding that MSC-EXOs have an obvious inhibitory effect toward different effector cells involved in the innate and adaptive immune response. Moreover, substantial progress has been made in the treatment of autoimmune diseases, including multiple sclerosis (MS), systemic lupus erythematosus (SLE), type-1 diabetes (T1DM), uveitis, rheumatoid arthritis (RA), and inflammatory bowel disease (IBD). MSC-EXOs are capable of reproducing MSC function and overcoming the limitations of traditional cell therapy. Therefore, using MSC-EXOs instead of MSCs to treat autoimmune diseases appears to be a promising cell-free treatment strategy. In this review, we review the current understanding of MSC-EXOs and discuss the regulatory role of MSC-EXOs on immune cells and its potential application in autoimmune diseases.

## 1 Introduction

Mesenchymal stem cells (MSCs) are pluripotent stem cells with the capacity for self-renewal and multidirectional differentiation into osteoblasts, chondrocytes, adipocytes, and other types of cells (1, 2). MSCs are widely distributed in the body and have been isolated from a variety of tissues, among which the bone marrow and subcutaneous fat are common cellular sources (3). The International Committee established the recognition characteristics of human MSCs, including under standard culture conditions to maintain adhesion appearance, expression of CD105, CD73, and CD90 molecules, no expression of CD45, CD34, CD14, CD45, CD11b, CD79a, CD19, and HLA-DR, with the ability to differentiate into osteoblasts, adipocytes, chondrocytes *in vitro* (4). In addition to its strong differentiation capacity, MSCs also have immunomodulatory potential to modulate innate and adaptive immune cells (5). Abundant evidence indicates that MSCs can act on natural killer (NK) cells, dendritic cells (DCs), macrophages, B lymphocytes, and T lymphocytes *via* inhibiting the activation, proliferation and differentiation into effector cells (6–9). Following stimulation with inflammatory factors, MSCs exhibit the properties of reducing the inflammatory response, improving tissue repair, and avoiding infection by secreting various immune regulatory factors (10). At present, substantial evidence suggests that MSCs exert their immunomodulatory function through paracrine pathway, especially *via* exosomes (10, 11).

Extracellular vesicles(EVs) are a family of particles/vesicles found in blood and body fluids. They are composed of phospholipid bilayer and carry a variety of molecules that serve as mediators for intercellular communication (12). In 1946, Chargaffaf et al. suspected the existence of EVs and published the first study of EVs (13). It was not until 1967 that Wolf et al. confirmed the existence of EVs with electron microscopy (EM) (14). The term exosome (“ exo ”= external,“ some ”= body) was introduced in the 1970s, and it wasn’t until 1981 that the term“ exosome ”was first used by Trams to refer to Evs (15). Shortly after Johnstone et al. described multivesicular bodies (MVBs) and their 40-80nm exosomes in 1985 (16), differential centrifugation and ultracentrifugation above 100,000 g were used to separate and distinguish the smallest vesicles (17). With the increase in EVs (especially exosomes) publications, most studies do not clearly distinguish exosomes from other vesicles. To address this problem, in 2013, the International Extracellular Vesicle Society (ISEV) proposed a set of criteria for EV Science (18). In order to ensure normative research, the Minimal Information of Studies of Extracellular Vesicles (MISEV) was revised in 2018 to update knowledge in the field (19).

Exosomes are spherical vesicles composed of lipid bilayer membranes with a diameter of 40-200 nm, which contain complex and abundant active substances such as proteins and nucleic acids. Expression of exosome markers, proteins, nucleic acids, and other bioactive molecules is related to the cell of origin (20). There is increasing evidence that MSC-derived exosomes (MSC-EXOs) play an important role in immune regulation. MSC-EXOs are spherical vesicles secreted by MSCs that contain many anti-inflammatory compounds and modulate the immune response by interacting with immune effector cells (12). In the treatment of autoimmune diseases, MSC-EXOs as a carrier of cell-free therapy have attracted extensive attention, because they not only carry most of the therapeutic effects of MSC itself, but also reduce the concerns about the safety of injecting live cells. MSC-EXOs has significant advantages over MSCs in clinical treatment and may completely replace MSC therapy in the future.

## 2 Characteristics of Mesenchymal Stem Cell-Derived Exosomes

**Figure 1:**
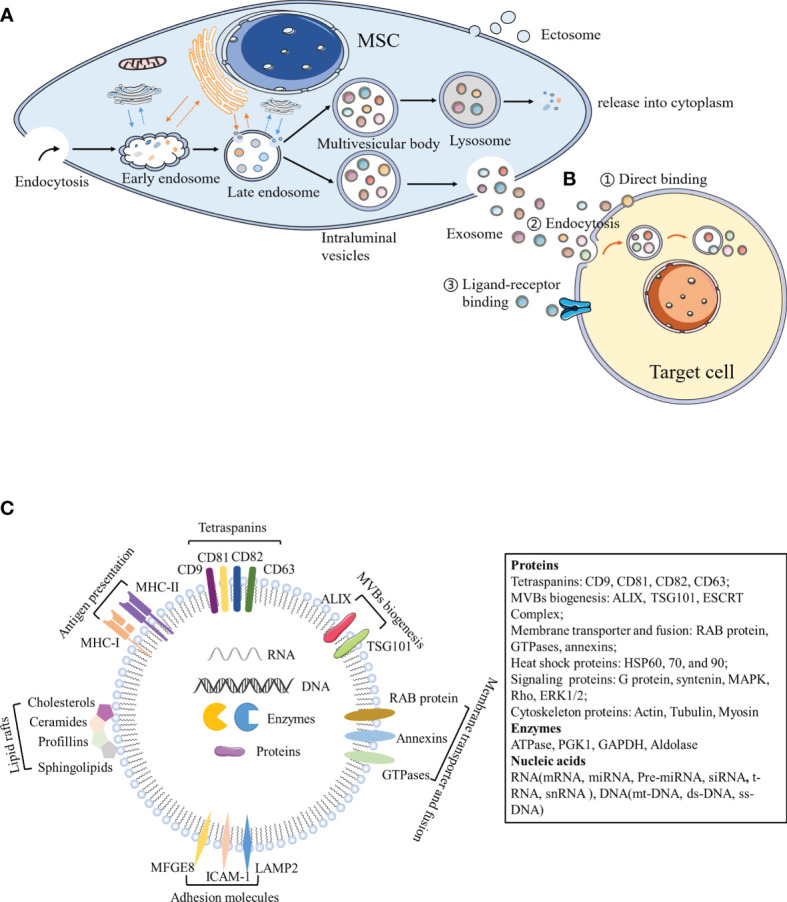
Biogenesis and components of exosomes. **(A)** Exosomes originate from the endosomal pathway. Extracellular material enters the cytoplasm through plasma membrane depression and endocytosis, and fuses with early endosomes, endoplasmic reticulum and preformed Golgi bodies, to develop into late endosomes, which are interlinked with the cell membrane network structure to form ILVs containing a vesicle structure. Different concentrations and sizes of ILVs constitute MVBs. On the one hand, MVBs fuse with lysosomes, degrade the contents, and release them into the cytoplasm. On the other hand, MVBs are transferred to the cytoplasmic membrane through the membrane system and vesicles are released outside the cell, which are termed exosomes. **(B)** Exosomes can act by binding to receptors present on the surface of target cells, by binding to endocytosis, or by the direct binding to recipient cells. **(C)** Exosome components. MFGE8, milk fat globule-EGF factor 8 protein; ICAM-1, intercellular adhesion molecule 1; MHC I and II, major histocompatibility complex I and II; LAMP2, lysosomal-associated membrane protein 2; MAPK, mitogen-activated protein kinase; ERK, extracellular signal-regulated kinase; PGK1, phosphoglycerate kinase 1; GAPDH, glyceraldehyde 3-phosphate dehydrogenase.

Both eukaryotic and prokaryotic cells release EVs, which are regarded as a part of their normal physiology and acquired abnormalities (20). EVs represent an important substance for intercellular communication. They participate in normal physiological processes, as well as play a critical role in disease occurrence and progression. Numerous experimental and clinical studies have shown that the immunomodulatory effects of MSCs can be primarily attributed to MSC-derived extracellular vesicles (MSC-EVs) (21). Although the classification of EVs is constantly evolving, it is generally accepted that EVs are classified into three main categories according to their size and biogenesis: apoptotic bodies (ABs), microvesicles, and exosomes (22–24). ABs (greater than 1,000 nm in diameter) are comprised of relatively large fragments of cells containing organelles derived from the cells undergoing apoptosis, which are transferred to phagocytes (25, 26). Microvesicles (100–1,000 nm in diameter) and exosomes (40–200 nm in diameter) belong to EVs at the nano level. Microvesicles, also known as ectosomes or microparticles, are released into the extracellular environment after directly budding or shedding from the plasma membrane (27). Exosomes originate from the endosomal pathway. Extracellular material fuses with early endosomes (ESEs) through membrane invagination and endocytosis, and gradually matures and develops into late endosomes (LSEs). The invagination of LSEs leads to the formation of intraluminal vesicles (ILVs), and multiple vesicles assemble to form MVBs, which fuse with the cell membrane and are subsequently released (**Figure 1**) (20). With regards to the delivery of exosome contents, exosomes can be bound by target cells through multiple pathways, of which the main mechanisms include endocytosis, ligand-receptor binding, or direct binding (**Figure 1**) (28–31). Exosomes are formed by budding through the endosome pathway and are wrapped in a lipid bilayer, contents of which can be protected by the external environment to maintain exosome integrity.

Exosomes contain a large number of proteins, lipids, transcription factors, as well as DNA, mRNA, and miRNA (32, 33). Exosome membranes contain lipid raft structures composed of cholesterol, spingomyosin, and ceramide and the tetraspanin protein family (CD63, CD81, and CD9) as an exosome marker (34). Exosomes also contain other common proteins, including MVB biogenesis proteins (Alix, TSG101, and ESCRT Complex), membrane transporter and fusion proteins (RAB protein, GTPases, and annexins), heat shock proteins (HSP60, 70, and 90), lipid related proteins, and phospholipases (35). Notably, MSC-EXOs express not only common surface markers such as CD81 and CD9, but also mesenchymal stem cell surface markers (CD44, CD73, and CD90) by flow cytometry (34). Proteomic analysis of exosomes isolated from human bone marrow-derived mesenchymal stem cells (hBM-MSC)provided evidence of 730 functional proteins associated with MSC proliferation, adhesion, migration, and morphogenesis (36). Surprisingly, protein packaging in exosomes is not random, because human primed MSCs secrete exosomes (pMEX), compared to human primed MSCs (pMSC), has a high concentration of specific subcategories of proteins, including secretory proteins and extracellular matrix (ECM) associated proteins, which may provide the molecular basis for its unique functional properties (37). In addition to proteins, MSC-EXOs also contain numerous RNA. Interestingly, RNA is specifically incorporated into exosomes, where it accumulates and then enters the recipient cell to do its job (38). Interestingly, enrichment of MSC-EXOs with network-informed miRNA further enhanced the intrinsic ability of MSC-EXOs to prevent apoptosis, promote angiogenesis and induce myocardial cell proliferation (39). MSC-EXOs change the activity and function of target cells primarily through the horizontal transfer of these substances (**Figure 1**). Due to the lack of specific markers, MSC-EXOs are currently prepared according to size or density. Most laboratories separate exosomes from conditioned media *via* hypercentrifugation, which cannot differentiate exosomes from other EVs or biological macromolecules (40). The MISEV2018recommends using the generic term “small/medium/large EVs” based on its size or density, rather than the classic terms “exosome”, “vesicle”, and “apoptotic body” (19). However, most of the current articles continue to use classic terms. The sizes of small/medium/large EVs are partially overlapped and cannot be strictly distinguished. Therefore, this review takes a cautious attitude towards the absolute definition of different types of vesicles, and focuses on the effects of nanoscale EVs (e.g., exosomes on immune cells) and various autoimmune diseases.

Numerous studies have shown that MSC-EXOs exhibit similar functions to that of MSCs (e.g., repairing damaged tissues, regulating immune responses, and playing anti-inflammatory effects) (10, 41). Although MSC therapy is widely regarded as an effective therapy for several immunological diseases, the direct therapeutic effect of MSCs remains limited. Due to the relatively large size of MSCs, intravascular administration may lead to vascular obstruction, which can result in pulmonary embolism and death in severe cases (42). In addition, allogenic immune rejection or abnormal chromosomal differentiation may occur during *in vivo* transplantation, and even malignant tumors may form (43). Also, MSCs cells age rapidly and are expensive to have a large-scale production (44). Using MSC-EXOs in humans has several potential advantages over MSCs. First, nano-scale exosomes are free to pass through various biological barriers without blocking microvascular circulation. Second, their application effectively prevents the metastasis of DNA-mutated cells and hinders the tumor development. Third, the number of MSCs decreases rapidly following transplantation, whereas the delivery of MSC-EXOs can continue to function in the body (10). In addition, MSC-EXOs are considered to be non-immunogenic and can be produced in a large-scale production for clinic application (44). However, toxicological studies of nanoparticles *in vivo* still need to be fully evaluated, particularly following long-term exposure (45). Moreover, the discovery of a broad therapeutic effect of exosome-mediated MSCs eliminates many of the challenges associated with the use of living replicative cells, as it fundamentally shifts living-cell-based MSCs therapy to a “cell-free” treatment, reducing the risk of living cell therapy. Therefore, MSC-EXOs, as cell-secreted natural EVs, has the advantage of being an ideal nanoscale drug carrier.

## 3 Immunomodulatory Function of Mesenchymal Stem Cell-Derived Exosomes

### 3.1 Innate Immunity

#### 3.1.1 Macrophages

**Figure 2:**
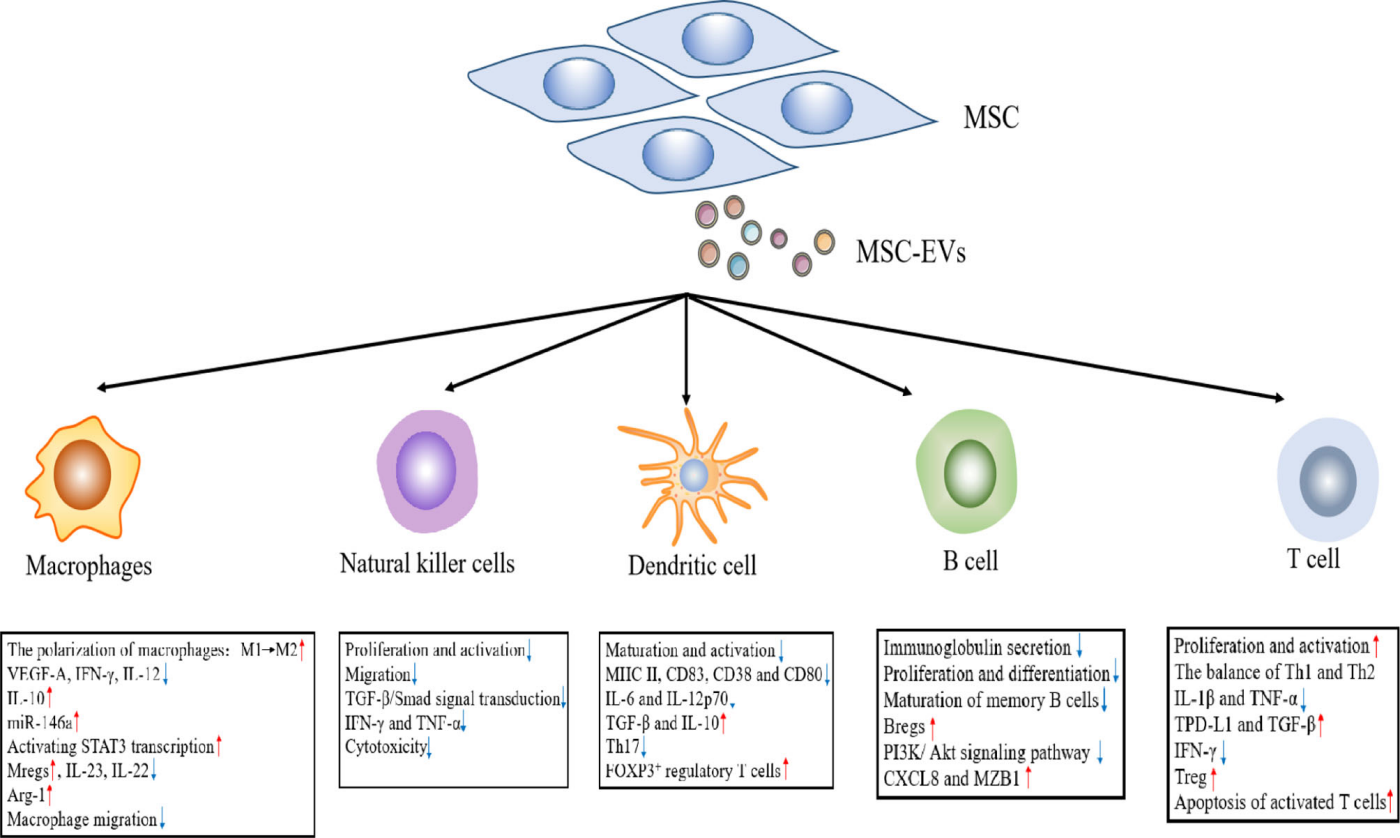
The effects of MSC-EVs on immune effector cells.

As an important aspect of the innate immune system, macrophages originate from either the yolk sac during embryonic development or bone marrow-derived monocytes (46). Under microenvironmental activation, macrophages may evolve into an M1 phenotype of pro-inflammatory macrophages or an M2 phenotype of anti-inflammatory macrophages. In general, M1 macrophages secrete pro-inflammatory molecules, including TNF-α and IL-1β, whereas M2 macrophages secrete immune regulatory factors (e.g., IL-10) (47). Recent data support the findings that the anti-inflammatory effect of MSC-EVs is inseparably related to macrophage polarization. MSC-EVs inhibit pro-inflammatory M1 macrophage activation and promote their polarization to M2 macrophages, which are consistent with a decrease in the levels of VEGF-A, IFN-γ, IL-12, and TNF-α, and an upregulation of IL-10 (48, 49). Previous studies have reported that macrophages are the main target cells for MSC-EVs to alleviate colon inflammation. In dextran sodium sulfate (DSS)-induced colitis, MSC-EVs effectively alleviate colitis by inducing an immunosuppressive M2 phenotype exhibited by colonic macrophage polarization. Compared with the control group, MSC-EV-treated mice produced a greater number of IL-10-producing M2 macrophages (49). Similarly, in a study of sepsis conducted by Song et al., the authors demonstrated that MSC-EVs promoted M2 macrophage polarization. Human umbilical cord-derived MSCs(hUC-MSCs) pretreated with IL-1β effectively induce macrophage polarization into an anti-inflammatory M2 phenotype *via* exosomal miR-146a, which ultimately resulted in prolonging the survival of sepsis mice (50). Moreover, the results showed that MSC-EVs down-regulated the production of IL-23 and IL-22, enhanced the anti-inflammatory phenotype of mature human regulatory macrophages (Mregs), which led to a weakened Th17 response (51). Zhao et al. reported that adipose-derived MSC-EXOs(AD-MSC-EXOs) promoted an M2 macrophage polarization by activating STAT3 transcription, up-regulating the expression of IL-10 and Arg-1 in macrophages, thereby inhibiting the inflammatory response of macrophages (52). In addition, in the rat model of experimental autoimmune uveitis (EAU), Bai et al. demonstrated that MSC-EXOs treatment downregulated the proportion of CD68^+^ macrophages in the retina and demonstrated the down-regulation of MSC-EXOs on macrophage migration to the retina (**Figure 2**) (53). Interestingly, MSC-EXOs also enhanced the immunosuppressive function of macrophage precursor, which is called myeloid-derived suppressor cells (MDSCs), a heterogeneous population of immature Myeloid derived cells. IL-6 secreted by olfactory ecto-mesenchymal stem cell-derived exosomes (OE-MSC-EXOs) activates the JAK2/STAT3 pathway in MDSCs, and enhances the inhibitory function of MDSCs by upregulating the levels of arginase, ROS and NO (54). In addition, abundant S100A4 in OE-MSC-EXOs mediated endogenous IL-6 production of MDSCs through TLR4 signaling, and along with exosomal-derived IL-6 promotes the immunosuppressive function of MDSC (54).

#### 3.1.2 Natural Killer Cells

NK cells are important effector cells in the innate immune response and play an important role in the host pathogen defense. When pathogens invade a host, they kill target cells by releasing cytotoxic particles containing perforin and granzyme (55). Although autoimmune diseases are primarily caused by T and B lymphocytes, NK cells possess excessive activation and inhibitory receptors, which can play a role in regulating autologous cell reactivity (56). In addition, NK cells may regulate the activity of other immune cells by secreting cytokines and influence the development of the adaptive immune response through these pathways (57). MSC-EVs primarily induce an immunosuppressive effect on NK cells, including the proliferation, activation, and cytotoxicity of NK cells. In a rat model of EAU, an injection of MSC-EXOs around the eye restored EAU damage by downregulating the transport of CD161^+^ NK cells in the lesion (53). Recent studies have shown that human fetal liver (FL) MSC-derived exosomes(hFL-MSC-EXOs) mediated downstream TGF-β/Smad signal transduction through the surface expression of TGF-β to inhibit the proliferation and activation of NK cells (58). In human graft-versus host disease (GVHD) experiments, researchers noted that MSC-EXOs suppressed the release of IFN-γ and TNF-α by activated NK cells, which reduced the cytotoxic effect of NK cells, and lessened the inflammatory response (**Figure 2**) (59).

#### 3.1.3 Dendritic Cells

DCs play an immunological role as antigen presenting cells (APCs), which can ingest, process, and present antigens to T and B lymphocytes (60). Most DCs in the body are immature DCs (iDCs) under normal circumstances and express low levels of class II major histocompatibility complex (MHC II) and T cell costimulatory molecules (CD80, CD86, and CD40). In addition, iDCs up-regulate the surface expression of MHC II and costimulatory molecules after ingesting antigens or cytokine stimulation, and are converted into mature DC (mDCs) (61). Previous studies indicate that the immunosuppressive effect of MSC-EVs on DCs is primarily realized by inhibiting DC maturation and activation. In the presence of MSC-EVs, iDCs are impaired in their ability to receive antigens and differentiate mDCs, which results in the decreased expression of mature and activation markers (e.g., CD83, CD38 and CD80), and correspondingly decreased the secretion of pro-inflammatory cytokines (i.e., IL-6 and IL-12p70) (62). At the same time, MSC-EVs can also enhance the release of TGF-β and IL-10 in CD11c^+^ DCs, thereby inhibiting lymphocyte proliferation (63). Researchers suggest that MSC-EVs treatment inhibits cell surface expression of MHC II and costimulatory molecules on CD11c^+^ cells in a dose-dependent manner. The results showed that DCs exhibited a hypoactive phenotype and thereby suppressed the subsequent T cell activation and proliferation (64). Moreover, the co-culture of MSC-EV-treated DCs with T cells reduced Th17 cell counts and IL-17 levels, and increased Foxp3^+^ regulatory T cells. This finding is consistent with the previous conclusion (65). In brief, there was a weakened ability of iDCs co-cultured with MSC-EXOs to differentiate into mDCs. This led to an inability to perform antigen presentation and effectively stimulate T lymphocyte proliferation. In contrast, it promoted an increase in Treg cells, which indirectly reflected that MSC-EXOs can enhance host immune tolerance (**Figure 2**).

### 3.2 Adaptive Immunity

#### 3.2.1 B Cells

B cells are important adaptive immune cells, whose main functions are to mediate humoral immunity and secrete antibodies. The regulation of MSC-EVs on B lymphocytes was investigated *in vitro*. In the CpG-stimulated PBMC co-culture system, MSC-EVs can completely replicate the inhibitory effect of MSCs on immunoglobulin secretion, B cell proliferation and differentiation in a dose-dependent manner (66, 67). Budoni et al. also claimed that MSC-EVs were internalized by activated CD19^+^/CD86^+^ B cells to inhibit B cell proliferation, differentiation, and antibody production, as well as inhibit memory B cell maturation (68). There are functional B cell subsets, known as regulatory B cells (Bregs). The key function of Bregs is to release IL-10, which inhibits the production of proinflammatory cytokines and supports regulatory T cell differentiation (69). MSC-EVs exert dose-dependent anti-inflammatory effects by inhibiting B cell maturation and inducing Bregs in lymph nodes in a murine model of collagen-induced arthritis (CIA) and delayed-type hypersensitivity (DTH). Moreover, MSC-EVs also regulate cellular function through the differential expression of mRNA in related genes. Researchers found that MSC-EVs induced B cells to down-regulate the PI3K/Akt signaling pathway through miR-155-5p, inhibited B cell proliferation and reduced the activation capacity of B lymphocytes (70). Real-time PCR analysis confirmed that following exosome treatment from MSC sources, the expression of genes that played an important role in B cell immune regulation (e.g., CXCL8 and MZB1) were upregulated (67). These studies confirmed that MSC-EXOs can play an immunomodulatory role by acting on B cells (**Figure 2**).

#### 3.2.2 T Cell

T cell proliferation and activation contribute to the occurrence and development of many autoimmune diseases. MSC-EVs play a negative role in T cell proliferation and activation. MSC-EVs have been shown to carry a variety of active molecules, including TGF-β (68, 71), IDO protein (72), and miR-125a-3p (73). These molecules give MSC-EVs the ability to inhibit T cell proliferation and activation. Studies have revealed that adenosine is associated with a robust immunosuppressive effect, and MSC-EVs inhibits T cell proliferation *in vitro* through adenosine signal transduction (74). MSC-EVs can also adjust the balance of T helper type 1 (Th1) and T helper type 2 (Th2) cells and reconstruct the stable state of Th1 and Th2. Chen et al. co-cultured bone marrow mesenchymal stem cell (BM-MSC)-derived exosomes (BM-MSC-EXOs) and peripheral blood mononuclear cells. The authors subsequently found that it could promote the Th1 to Th2 conversion of helper T cells, significantly reduced the levels of pro-inflammatory factors, IL-1β and TNF-a, and improved the levels of anti-inflammatory TGF-β (66). In a similar study, human adipose tissue MSC-EXOs inhibited the differentiation and activation of T cells and the release of pro-inflammatory factors (e.g., IFN-γ) (75). In addition, MSC-EXOs can facilitate the differentiation of Tregs. Treg cells are immune cells with negative feedback regulation. Similarly, inflammatory IL-1β-primed MSC-EVs upregulated PD-L1 and TGF-β expression, which induced the apoptosis of activated T cells, and increases the proportion of Treg cells in a mouse model of autoimmune encephalomyelitis (76). Previous studies in our laboratory have also found that OE-MSC-EXOs had a potent inhibitory effect on the proliferation of CD4^+^ T cells in experimental colitis mice. At the same time, the secretion of IL-17 and IFN-γ by T cells was reduced, whereas the secretion of TGF-β and IL-10 was enhanced, which could significantly slow disease progression (77). Following treatment with OE-MSC-EXOs, the Th1/Th17 subsets were significantly reduced, while Treg cells were increased (77). It was found that transgene-free human induced pluripotent stem cells (iPSCs) derived EVs could prevent the progression of Sjögren’s syndrome (SS) by inhibiting the differentiation of follicular helper T (Tfh) and Th17 cells (78). In addition, other studies have reported that MSC-EXOs can promote the differentiation of monocytes into an M2 macrophage phenotype, thereby activating the differentiation of CD4^+^ T cells into Treg cells and delaying the occurrence of immune rejection (**Figure 2**) (79).

## 4 Applications of Mesenchymal Stem Cell-Derived Exosomes in Autoimmune Diseases

To develop EVs as a cell-free therapy and elucidate its potential role in stem cell effects *in vivo*, the following contents can assess the role of exosomes released by MSCs in the treatment of autoimmune diseases, including Multiple sclerosis (MS), Systemic lupus erythematosus (SLE), Type-1 diabetes (T1DM), uveitis, Rheumatoid arthritis (RA), and Inflammatory bowel disease (IBD) (**Table 1**).

**Table 1:** The role of MSC-EVs in the treatment of autoimmune diseases, as discussed in the text.

Disease types	Animal model	Exosomes sources	Exosomes reactive molecules	Target cells/tissue	Mechanism of action	Effect	Ref
Multiple sclerosis (MS)	Experimental autoimmune encephalomyelitis (EAE)	BM-MSCs	Immune response/inflammatory response/myellination-related proteins (unspecified)	Microglia	The polarization of microglia from M1 to M2	Relieves the neurobehavioral symptoms and attenuates the inflammation and demyelination of CNS	(85)
		BM-MSCs	PD-L1, GAL-1 and TGF-β	Lymphocyte	Inhibits the activation and proliferation Promotes the secretion of anti-inflammatory cytokines	Alleviates the disease progression	(76)
		hPDLSCs	IL-10 and TGF-β	Spinal cords	NALP3 inflammasome inactivation, and NF-kB reduction	Attenuates the inflammation Infiltration	(86)
		hBM-MSCs	Anti-inflammatory RNAs, anti-inflammatory and neuroprotective proteins(unspecified)	Microglia	Infiltration of microglia reduces The formation of Treg	Alleviates the development of EAE	(89)
Type-1 diabetes (T1DM)	Nonobese diabetic (NOD)	BM-MSCs	unspecified	Neurons and astrocytes	Repairs damaged neurons and astrocytes	Improves cognitive impairment	(100)
		AD-MSCs	unspecified	T/B lymphocytes	Increases the expression of anti-inflammatory factors and the population of Treg cells	Prevents overactivation and autoimmune damage	(101)
		hBM-MSCs	unspecified	Islet cells	Inhibits islet inflammation	Increases the plasma insulin level	(64)
Uveitis	Experimental autoimmune uveitis (EAU)	hBM-MSCs	unspecified	Th1 and Th17 cells	Decreases the number of Th1 and Th17 cells	Inhibits the development of EAU	(64)
		hUC- MSCs	unspecified	CD3^+^Tcells Macrophages	Decreases CD3^+^T cells infiltration and macrophages migrating to the retina	Alleviates the development of EAU	(53)
		hBM-MSCs	unspecified	CD4^+^T cells	Induces the transformation of CD4^+^T to Treg cells	Plays immunomodulation function on EAU	(64)
Rheumatoid arthritis (RA)	Collagen-induced arthritis (CIA)	BM-MSCs	miRNA-150-5p	Fibroblast-like synoviocytes (FLS)	Targets MMP14 and VEGF.2	Decreases migration and invasion in RA FLS and downregulates tube formation in HUVECs	(112)
		BM-MSCs	miRNA-320a	FLS	Suppresses CXCL9 expression	Attenuates arthritis and bone damage	(113)
		BM-MSCs	miRNA-192-5p	FLS	Inhibits the levels of pro-inflammatory factors and suppresses synovial hyperplasia by RAC2	Delays the event of the inflammatory response	(114)
		BM-MSCs	unspecified	B cell	Expands Breg cells and decreases plasmablast differentiation	Lowers disease incidence and deceases clinical score Reduces levels of serum auto-antibodies	(48, 115)
Sjögren’s syndrome (SS)	Experimental Sjögren’s syndrome (ESS)	OE-MSCs	IL-6	MDSC cells	Activates the Jak2/Stat3 pathway in MDSCs	Upregulates arginase expression and increases ROS and NO levels attenuates disease progression	(54)
		BM-MSCs and iPSC-MSCs	unspecified	SG epithelial cells (SGECs) and immune cells	Inhibits the differentiation of Tfh and Th17 cells and the activation of APCs	Decreases the lymphocyte infiltration in salivary glands and serum autoantibody levels	(78)
Inflammatory bowel disease (IBD)	Experimental Colitis	hUC -MSCs	unspecified	Macrophages	Inhibits the expression of iNOS and IL-7	Relieves inflammatory responses, and attenuates DSS induced colitis	(121)
		hUC -MSCs	miR-326	Human colorectal mucosa cells (FHC)	Targets the expression of NEDD8 (neural precursor cell-expressed, developmentally downregulated gene 8)	Inhibits the neddylation process and achieves the effect of relieving IBD	(122)
		BM-MSCs	miR-146a	Colonic epithelial cells	Inhibits TNF receptor-associated factor 6 (TRAF6) and IL-1 receptor-associated kinase 1 (IRAK1) expression	Ameliorates the disease severity	(123)
		hUC -MSCs	TSG-6	Th2 cells Th17 cells	Enhances the immune response of Th2 cells in MLN and reduces the immune response of Th17 cells	Ameliorates IBD symptoms and reduces mortality rate	(119)
		OE-MSCs	unspecified	Th1/Th17 cells Treg cells	Regulates Th-cell responses	Alleviates the severity of disease	(77)
		AD-MSCs	unspecified	Treg cells	Regulate the Treg population	Improves inflammation in DSS‐induced acute colitis	(124)
		hBM -MSCs	Metallothionein-2	Macrophages	Polarizes M2b macrophages	Attenuates mucosal inflammation	(125)

### 4.1 Multiple Sclerosis

MS is the most common inflammatory disease of the central nervous system (CNS), which is characterized by demyelination, neuronal damage and loss, and ultimately neurological dysfunction (80). Experimental autoimmune encephalomyelitis (EAE) is the commonly used animal model of MS (81). As the primary immune cells of the central nervous system, microglia can maintain tissue homeostasis and contribute to central nervous system development under normal physiological conditions (82). Following pathogen invasion, activated microglia with an M1 phenotype as the first line of defense secrete pro-inflammatory cytokines to eliminate invading pathogens. However, when tissue homeostasis is restored, microglia exhibiting an M2 phenotype need to be activated, otherwise the excessive release of pro-inflammatory cytokines can lead to neuronal damage (83). Microglia have both neurodestructive and neuroprotective functions. An imbalance of the M1/M2 phenotype inhibits the nerve protection function and promotes the occurrence of MS (84). Therefore, M1 to M2 polarization of microglia may be beneficial to the neuroprotective function of microglia, thereby preventing disease progression. The study by Zijian et al. demonstrated that BM-MSC-EXOs therapy inhibits microglia from developing into an M1 phenotype, promotes M2 phenotype polarization, and secretes anti-inflammatory cytokines (e.g., TNF-α, IL-10, and TGF-β). Moreover, BM-MSC-EXOs treatment significantly improved the neurobehavioral symptoms of EAE rats and relieved the inflammation and demyelination of CNS (85). Human periodontal ligament stem cells (hPDLSCs)-EVs from MS patients has been shown to inhibit NALP3 inflammasome activation in an EAE model (86). Although substantial progress has been made in the treatment of MS, the appropriate treatment for MS remains controversial. The currently available drugs may have potentially harmful side effects and cannot meet future needs (87). MSC-EXOs is a natural non-toxic vesicle that can deliver mRNA, miRNA, and proteins, as well as alleviate the condition of EAE by regulating microglia polarization (88). As the carrier of MSC-specific tolerance molecules (e.g., PD-L1, Galecin-1 [GAL-1], and TGF-β), MSC-EVs can effectively inhibit the activation and proliferation of self-responding lymphocytes and promote the secretion of anti-inflammatory cytokines derived from lymphocytes, thereby alleviating EAE disease progression (76). In another study, it was also shown that the infiltration of microglia in spinal cord sections treated with MSC-EXOs was significantly reduced, and MSC-EXOs induced the formation of Tregs and alleviated the development of EAE (89). Therefore, MSC-EXOs may hold great potential for the treatment of MS.

### 4.2 Systemic Lupus Erythematosus

SLE is a chronic autoimmune disease characterized by immune inflammation and multiple organ damage. In addition, SLE pathogenesis is extremely complex, primarily due to the comprehensive influence of genetic, environmental, hormonal, epigenetic, and other factors (90). With the help of Tfh cells, anti-nuclear antibodies (ANA) are produced, leading to the deposition of immune complexes in vital organs. The immune complex triggers an influx of large inflammatory cells by activating the complement cascade, causing tissue inflammatory damage (e.g., nephritis) (91). Nephritis represents the leading cause of morbidity and mortality in SLE and occurs in approximately half of all patients (92). In previous studies, researchers found that the infusion of hBM-MSCs into mouse models of lupus nephritis reduced the level of autoantibodies, improved the survival rate in mice, and alleviated the clinical symptoms of glomerulonephritis by inhibiting the development of Tfh (91). Moreover, the loss of BM-MSC/osteoblast function in an SLE mouse model results in an impairment of the osteoblastic niche and an imbalance of immune homeostasis. Allogeneic bone marrow mesenchymal stem cell transplantation (MSCT) plays a positive role in reconstructing the osteoblast niche and restoring immune homeostasis; thus, effectively reversing multiple organ dysfunction (93). The above findings confirm a positive therapeutic effect of MSCs on SLE; however, cellular therapy continues to face technical, cost, and regulatory challenges in clinical trials. Recent studies have demonstrated that the immunoregulatory activity of MSCs was mainly mediated by paracrine factors (e.g., MSC-EVs) (66). In a novel porcine model of coexisting metabolic syndrome (MetS) and renal artery stenosis (RAS), MSC-EVs isolated from adipose tissue were capable of improving renal structuring and function, and reducing renal injury and dysfunction by up-regulating the level of IL-10 expression (94). Although MSC-EVs have been observed to inhibit autoimmune diseases *in vitro*, there have been no studies on the use of MSC-EVs for the treatment of SLE in mice or humans. In the future, MSCs are expected to provide a novel and safe treatment for SLE patients.

### 4.3 Type-1 Diabetes

T1DM is considered a chronic autoimmune disease that is influenced by genetic, immune, and environmental factors (95). T1DM is primarily caused by the autoimmune destruction of beta-cells in the islets of Langerhans, leading to insufficient insulin secretion (96). Non-obese diabetic (NOD) mice are the preferred spontaneous disease model of T1DM. NOD mice exhibited the same clinical symptoms of diabetes as human beings-hyperglycemia, polyuria, and polydipsia (95). Although insulin therapy remains the main treatment method in the short term, MSCs have recently attracted wide attention as a promising method for the treatment of diabetes (96). MSCs display immunomodulatory properties in inflammatory diseases, and their immunomodulatory effects have been studied in T1DM (97, 98). Human MSCs have the ability to delay the onset of autoimmune diabetes by inhibiting the development of Th1 cells, which may improve the efficacy of islet transplantation in patients with T1DM (99). Currently, it is generally believed that a paracrine mechanism of action is more direct in MSCs *in vivo*, particularly MSC-EXOs (10). In previous studies, the effect of MSC-EXOs on diabetes has been investigated. Previous studies have shown that exosomes released by BM-MSCs had similar functions to those of BM-MSCs, and they had the ability to improve cognitive impairment in diabetic mice by repairing damaged neurons and astrocytes (100). In another study, AD-MSC-EXOs were found to have a significant mitigatory effect on T1MD by increasing the expression of anti-inflammatory factors (e.g., IL-10), and the population of Tregs that are equipped to suppress the immune response, preventing immune overactivation and autoimmune damage (101). In addition, the results of the study by Shigemoto-kuroda et al. also confirmed that MSC-EVs could inhibit islet inflammation, significantly increasing the plasma insulin levels, and effectively delaying the occurrence of T1DM in mice (64). These results suggested that MSC-EVs have great potential as a cellular therapy for the prevention of T1DM.

### 4.4 Uveitis

Uveitis is the leading cause of visual disability worldwide. Uveitis can be divided into three categories according to etiology: 1) infectious uveitis; 2) non-infectious uveitis; and 3) masquerade uveitis, of which non-infectious uveitis is believed to be caused by autoimmunity, and EAU is used as a mouse model (102). Traditional immunosuppressive drugs (e.g., corticosteroids) and novel biological agents have been shown to be effective for the treatment of uveitis; however, side effects and unknown long-term safety often limit the use of these drugs (103). A large number of experimental results have suggested that MSC-EXOs have a positive effect on inflammatory eye disease. EAU mice that do not MSC-EXOs therapy have severely damaged retinal photoreceptors and the infiltration of inflammatory cells, whereas EAU mice with exosomes injected through tail vein displayed eyes similar to that of normal mouse retinas, with almost no structural damage and inflammatory infiltration (64). Compared with EAU mice treated with PBS, mice treated with MSC-EXOs exhibited a significant decrease in CD3^+^ T cells infiltrating the retina and a decrease in the number of macrophages migrating to the retina (53). In addition, Th1 and Th17 cells represent important pathogenic factors in the development of EAU disease. Flow cytometry results of the cervical draining lymph nodes (CLNs) showed that the number of IFN-γ^+^CD4^+^ cells (Th1) and IL-17^+^CD4^+^ cells (Th17) in EAU mice treated with MSC-EXOs was significantly lower than that in the PBS-treated mice (64). The above results indicate that MSC-EXOs can inhibit the development of EAU by inhibiting Th1 and Th17 cells. Part of the immunomodulatory function of MSCs on EAU is to induce the transformation of CD4^+^ T cells into antigen-specific CD4^+^CD25^+^Foxp3^+^ Tregs by secreting TGF-β in a paracrine manner (104). In another study, MSC-EVs alleviated EAU by directly inhibiting the development of Th1 and Th17 cells rather than inducing Tregs to inhibit T cell proliferation (64). Although there may be some differences between MSC-EVs and MSCs in various immunomodulatory pathways, the treatment of EAU using MSC-EVs also represents a potential new method.

### 4.5 Rheumatoid Arthritis

RA is a chronic inflammatory disease characterized by synovial hyperplasia and immune cell infiltration, leading to joint destruction (105, 106). The microvesicles derived from BM-MSCs carry the regulatory molecules that exist in the mother cell, including PD-L1, GAL-1, and TGF-β1 (76). As a PD-1 receptor, PD-L1 plays a key role in regulating the development of inducible T regulatory cells (iTregs) (107). In addition, GAL-1 is an endogenous lectin that has been shown to induce growth arrest and the apoptosis of activated T cells, as well as the immunoregulation mediated by regulatory T cells (108, 109). Researchers found that the injection of co-gene DBA/1 fibroblasts secreting GAL-1 inhibited the progression of arthritis through T cell apoptosis in collagen-induced arthritis (110).

Moreover, exosomal miRNA also plays an important role in alleviating the development of RA (111). For example, MSC-derived miRNA-150-5p-expressing exosomes decreased the migration and invasion in RA fibroblast-like synoviocytes (FLS) and downregulated tube formation in HUVECs by targeting MMP14 and VEGF (112). In 2020, researchers found that BM-MSCs-secreted exosomal miRNA-320a and miRNA-192-5p also acted on FLS, reducing inflammatory response and alleviating the progression of RA (113, 114). Despite the pathogenic role of B cells in RA, recent studies have demonstrated the therapeutic effect of MSC-EXOs *via* expanding Bregs (48, 115). BM-MSC-EXOs-treated CIA mice exhibited a lower disease incidence and deceased clinical score, accompanied by reduced levels of serum auto-antibodies (115). Furthermore, such phenomena was associated with decreased plasmablast differentiation and the generation of Bregs (48). Interestingly, similar therapeutic effects have been revealed in osteoarthritis (OA). The lncRNA KLF3-AS1 was significantly enriched in MSC-EXOs, which promoted cartilage repair and chondrocyte proliferation in OA rat models (116). In addition, in a model of porcine synovitis induced by bovine serum albumin, the intraarticular injection of BM-MSC-EVs into pigs had an anti-inflammatory effect, with a reduced number of synovial lymphocytes and down-regulated level of TNF-α transcription (117). These results provide evidence for a role of MSC-EVs for the treatment of inflammatory diseases (e.g., arthritis). MSC-EVs provide novel insight into the treatment of RA, which may lead to new therapeutic opportunities and strategies for RA.

### 4.6 Inflammatory Bowel Disease

IBD is a chronic, nonspecific, relapsing inflammatory gastrointestinal disease associated with mucosal immune system disorders and gastrointestinal injury. IBD, which primarily includes ulcerative colitis (UC) and Crohn’s disease (CD), has become a global disease with an increasing incidence (118). Studies of this disease have mainly used DSS and 2,4,6-trinitrobenzenesulfonic acid (TNBS) to induce IBD in mouse models (119). Existing drugs to treat IBD are still very limited, Therefore, there is an urgent need to develop safe and effective treatments for IBD (120). At present, several studies have shown that MSC-EXOs/exosome components have potential functions in the development of IBD and can serve as potential targets for the diagnosis and treatment of IBD. In a model of IBD induced by DSS, hUC-MSC-EXOs treatment decreased the infiltration of macrophages in colon tissue and inhibited the expression of IL-7 (121). hUC-MSC-EXOs also inhibited neddylation and alleviated IBD by miR-326 (122). Another study also showed that exosomal miRNA of MSCs, such us miR-146a, downregulated TNF receptor-associated factor 6 (TRAF6) and IL-1 receptor-associated kinase 1 (IRAK1) expression, inhibited pro-inflammatory cytokine and enhanced the expression of IL-10 (123).Moreover, tumor necrosis factor-α stimulated gene 6 (TSG-6), as detected by hUC-MSC-EXOs, regulated the immune response of Th2 and Th17 cells in mesenteric lymph nodes (MLN), down-regulated the levels of pro-inflammatory cytokines in colon tissue, and up-regulated the levels of anti-inflammatory cytokines to protect the intestinal barrier (119). Other tissue-derived MSC-EXOs showed similar efficacy to that of hUC-MSC-EXOs in the treatment of DSS induced IBD. OE-MSC-EXOs significantly improved the severity of experimental colitis in mice primarily by modulating the immune response of Th-cells, including a significant reduction in Th1/Th17 subsets and an increase in Treg cells (77). Neda Heidari et al. reported that AD-MSC-EXOs therapy could restore the percentage of Treg in the spleen to a baseline level similar to that in normal mice and improved inflammation in DSS induced acute colitis (124). Furthermore, metallothionein-2 in hBM-MSC-EXOs inhibited inflammatory responses, polarized M2b macrophages, and maintained intestinal barrier integrity (125). With the further research on exosomes, exosomal-structure design of novel drugs may provide new insights for IBD.

## 5 Conclusion and Prospects

The treatment of autoimmune diseases is challenging and there is currently no effective cure. In this review, we discussed the regulatory role of MSC-EXOs on immune cells and the new progress of MSC-EXOs in the treatment of autoimmune diseases, suggesting that MSC-EXOs may be a new cell-free drug for the treatment of autoimmune diseases.

In view of the therapeutic potential of MSC-EXOs in preclinical studies, there are currently 19 clinical trials looking at its application in a variety of diseases (Available online: http://www.clinicaltrials.gov/). Although encouraging results have been achieved with MSC-EXOs, several uncharted territories remain to be explored before MSC-EXOs can be used as drug vectors for clinical use. First, although it has been reported that clinical-grade exosomes can be produced using good manufacturing techniques and standard operating procedures, large-scale production of exosomes for clinical use remains to be explored (126). Second, support materials can be used to maximize the therapeutic power of MSC-EXOs. In recent years, hydrogel has attracted much attention among biocompatible auxiliary materials. In the experimental model of ischemia, the retention time of MSC-EXOs with an injectable hydrogel was prolonged, which enhanced the therapeutic effect of exosomes (127). In the experiment of preventing hyperplastic scar in rabbit ear model, adipose-derived stem cell conditioned Medium (ADSC-CM) was combined with polysaccharide hydrogel to prolong the therapeutic effect of cytokines (128). Third, in order to ensure the safety of patients treated with MSC-EXOs, the route and dose of exosomes must be explored. At present, the main route of administration in clinical studies is intravenous infusion. However, aerosol inhalation of MSC-EXOs was used in clinical trials to explore the efficacy of MSC-EXOs in severe pulmonary diseases (Clinical Trials. Gov Identifier: NCT04313647). In addition, the clinical trial assessing the safety and efficacy of MSC-EXOs in Patients with Alzheimer’s disease was conducted with nasal drip (ClinicalTrials.gov Identifier: NCT04388982). Therefore, the route of exosome administration needs to be determined according to the actual situation of the disease. In addition, clinical trials need to monitor patients treated with MSC-EXOs in real time to ensure that the smallest dose is most effective. Therefore, future work should focus on the combination of basic research on MSC-EXOs with emerging technologies to bring new breakthroughs for the treatment of autoimmune diseases.

## Author Contributions 

ZS, WH, and JL drafted and revised the manuscript. JT and SW discussed and revised the manuscript. KR conceived the topic and revised the manuscript. All authors contributed to the article and approved the submitted version.

## Funding

This work was supported by the National Natural Science Foundation of China (Grant Nos. 81971542, 82171771), Summit of the Six Top Talents Program of Jiangsu Province (Grant No. 2017-YY-006).

## Conflict of Interest

The authors declare that the research was conducted in the absence of any commercial or financial relationships that could be construed as a potential conflict of interest.

## Publisher’s Note

All claims expressed in this article are solely those of the authors and do not necessarily represent those of their affiliated organizations, or those of the publisher, the editors and the reviewers. Any product that may be evaluated in this article, or claim that may be made by its manufacturer, is not guaranteed or endorsed by the publisher.
